# A deep learning approach to predict blood-brain barrier permeability

**DOI:** 10.7717/peerj-cs.515

**Published:** 2021-06-10

**Authors:** Shrooq Alsenan, Isra Al-Turaiki, Alaaeldin Hafez

**Affiliations:** 1 Information Systems Department, College of Computer and Information Sciences, Princess Nourah bint Abdulrahman University, Riyadh, Saudi Arabia; 2 Information Technology Department, College of Computer and Information Sciences, King Saud University, Riyadh, Saudi Arabia; 3 Information Systems Department, College of Computer and Information Sciences, King Saud University, Riyadh, Saudi Arabia

**Keywords:** Chemoinformatics, Convolutional Neural Network (CNN), Blood Brain Barrier (BBB) permeability, Quantitative Structure-Activity Relationships (QSAR)

## Abstract

The blood–brain barrier plays a crucial role in regulating the passage of 98% of the compounds that enter the central nervous system (CNS). Compounds with high permeability must be identified to enable the synthesis of brain medications for the treatment of various brain diseases, such as Parkinson’s, Alzheimer’s, and brain tumors. Throughout the years, several models have been developed to solve this problem and have achieved acceptable accuracy scores in predicting compounds that penetrate the blood–brain barrier. However, predicting compounds with “low” permeability has been a challenging task. In this study, we present a deep learning (DL) classification model to predict blood–brain barrier permeability. The proposed model addresses the fundamental issues presented in former models: high dimensionality, class imbalances, and low specificity scores. We address these issues to enhance the high-dimensional, imbalanced dataset before developing the classification model: the imbalanced dataset is addressed using oversampling techniques and the high dimensionality using a non-linear dimensionality reduction technique known as kernel principal component analysis (KPCA). This technique transforms the high-dimensional dataset into a low-dimensional Euclidean space while retaining invaluable information. For the classification task, we developed an enhanced feed-forward deep learning model and a convolutional neural network model. In terms of specificity scores (i.e., predicting compounds with low permeability), the results obtained by the enhanced feed-forward deep learning model outperformed those obtained by other models in the literature that were developed using the same technique. In addition, the proposed convolutional neural network model surpassed models used in other studies in multiple accuracy measures, including overall accuracy and specificity. The proposed approach solves the problem inevitably faced with obtaining low specificity resulting in high false positive rate.

## Introduction

The blood-brain barrier (BBB) is a membrane that regulates solutes in circulating blood from penetrating the central nervous system (CNS). The BBB is responsible for regulating the transport of 98% of the compounds to the CNS (Martins et al., 2012). A fundamental task of pharmaceutical companies is to identify which substances penetrate the BBB when developing drugs that target the CNS (Pardridge, 1999). Identifying compounds that can penetrate the CNS is critical to synthesizing medications for the treatment of many neurological diseases, such as brain tumors, Parkinson’s disease, and Alzheimers (Miao et al., 2019; Bagchi et al., 2019; Desai et al., 2007; Chevalier, 2018). A compound’s passage to the CNS can be measured by its active or passive transport (Seddon et al., 2009).

Before the emergence of in silico approaches, quantitative structure-activity relationship (QSAR) studies were conducted experimentally using in vivo and in vitro methods. QSAR is a classification and regression method widely used to analyze the relationship between a chemical structure and its activities; multiple studies have developed QSAR models to predict BBB permeability (Li et al., 2005b; Guerra, Páez & Campillo, 2008; Martins et al., 2012; Brito-Sánchez et al., 2015; Yuan, Zheng & Zhan, 2018). These experiments were performed on living organs or through clinical trials. Although these methods are accurate, they are expensive and time consuming. This inefficiency motivated the development of in silico methods, which use computational approaches and machine learning (ML) algorithms to find and validate new compounds from a database of molecules (Wermuth et al., 2015). These methods generate more data and enable more automated modeling to support drug design research.

The study of BBB permeability can be formulated into a binary classification problem with two class labels: BBB+ and BBB−, representing compounds with high BBB permeability and low BBB permeability, respectively. Developing a classification model requires a complete understanding of the primary issues shared by most BBB permeability studies. These issues are related to algorithm, dataset, and features. Algorithm issues include overfitting and low accuracy scores when predicting compounds with low permeability (i.e., BBB−). Features issues are caused by the high dimensionality of QSAR datasets and include overfitting and poor generalization to unseen data (Ghaddar & Naoum-Sawaya, 2018). Dataset issues are due to imbalanced class label distribution in the BBB dataset, which affects the classifier’s ability to predict compounds classified as BBB-.

Throughout the years, various deep learning (DL) models have been developed to solve the problem of BBB permeability. DL variations other than feed-forward artificial neural network (FFDNN) models have not been thoroughly investigated (Wang et al., 2018; Miao et al., 2019; Garg & Verma, 2006; Guerra, Páez & Campillo, 2008). A common characteristic of multiple BBB studies is the achievement of high accuracy rates when predicting compounds with high permeability. However, it has been a constant challenge to obtain satisfactory results when predicting compounds with low permeability (Wang et al., 2018; Guerra, Páez & Campillo, 2008; Li et al., 2005b). Improving the specificity score will help decrease false positive rates, which usually result from obtaining predictions of high BBB+ and low BBB− (Yuan, Zheng & Zhan, 2018). The main reasons for this issue are the unavailability of compounds with low BBB permeability (BBB−) and the inability to handle class imbalance. Previous studies have never adequately addressed these problems to enhance the model fitting process.

In this paper, we investigate the potential of DL approaches to solve the problem of BBB permeability. The primary goal of this study is to improve the accuracy of BBB classification models. We focus not only on identifying high penetration compounds but also low penetration compounds. Convolutional neural networks (CNN) (Fukushima & Wake, 1991) are powerful DL models that have delivered remarkable results for many problems, such as image processing and text classification (Schmidhuber, 2015), but have not been used to predict BBB permeability. We investigate the applicability of state-of-the-art CNN architecture to the problem of classifying BBB permeability. Because the dataset is high dimensional, this study uses feature extraction methods to extract the most useful features. This paper makes the following contributions. First, it presents an enhanced FFDNN model to predict compounds’ BBB permeability. The model can be called enhanced because it is based on a broad overview of network hyper-parameters that substantially affect the classification task and improve specificity scores. Second, to the best of our knowledge, it is the first study to investigate the use of state-of-the-art CNN architecture to model the problem of BBB permeability. This study addresses the problem of low specificity scores obtained by most BBB classification models without compromising other accuracy measures. Finally, this research study applies kernel PCA (KPCA) as a feature extraction method in the problem of BBB permeability. We investigate the influence of KPCA’s non-linearity on the classifier’s performance and visualize the dataset’s transformation.

The rest of this paper is organized as follows. First, we summarize previous studies in the literature that have attempted to solve the problem of BBB permeability. Second, we present the research methodology, describing the preprocessing steps taken before developing the DL classification model. This includes descriptors calculations, dimensionality reduction, and oversampling of the minority class. Then, we present an empirical comparative analysis of the proposed models and widely used machine learning models; namely, XGboost, support vector machine (SVM), and random forest (RF).

## Literature Review

The BBB is a complex system that regulates the transport of compounds through the CNS. A compound’s penetration to the BBB is measured by LogBB which is defined as the logarithmic ratio of the concentration of compounds in the brain to compounds in the blood (Bradbury, 1993). BBB permeability can be measured with two approaches: active or passive transport (Seddon et al., 2009). The active transport of compounds can be determined by clinical phenotypes related to drug side effects and indications (Gao et al., 2017; Miao et al., 2019). However, the most widely used approach relies on predicting BBB permeability through passive diffusion based on chemical structure or physical features (Carpenter et al., 2014).

Throughout the years, many ML and DL algorithms were developed to model this classification task. Support Vector Machine (SVM) models have been adopted in many studies, because it delivered decent results in predicting the positive class (BBB+) (Martins et al., 2012; Li et al., 2005b; Gao et al., 2017; Kortagere et al., 2008; Wang et al., 2018). One of the early SVM models was developed by Zhang et al. (2008) who worked on a small dataset of 150 compounds. They obtained an accuracy of 82% in predicting compounds that can penetrate the BBB. Decision tree models were developed by  Castillo-Garit et al. (2017) and Suenderhauf, Hammann & Huwyler (2012), where the former obtained 89.29% specificity accuracy. Over the years, multiple regression and predictive models were proposed. Brito-Sánchez et al. (2015) developed Multiple Linear Regression (MLR) and Linear Discriminant Analysis (LDA). Their experiment showed that linear models outperformed nonlinear ones in the classifier performance.

DL models have been used in QSAR since the 1990s (Ma et al., 2015). Early attempts with shallow ANN were presented by  Garg & Verma (2006), Guerra, Páez & Campillo (2008) and Dorronsoro et al. (2004). These models were developed with a smaller dataset of known chemical compounds, so their achieved accuracy was not satisfactory. A more recent study with a larger dataset was proposed by  Wang et al. (2018). To improve the learning capability of the predictive models, they developed multiple ML and DL models with a dataset of 2350 compounds. Their analysis found that a multilayer perceptron (MLP) consensus model outperformed all their proposed single models. The consensus model reached a sensitivity accuracy of 99% but failed to obtain a specificity accuracy above 83.3%.  Miao et al. (2019) attempted to create a DL classification model that depends on drugs’ side effects and indications. Their study considered clinical side effects and indications rather than chemical descriptors to predict a compound’s permeability.

Researchers have established that only 2% of compounds can actually penetrate the CNS (Martins et al., 2012), making it a challenging task to predict compounds with low permeability.  Yuan, Zheng & Zhan (2018) and  Wang et al. (2018) emphasized the importance of improving the classifier’s specificity scores to avoid the occurrence of high numbers of false positives. Yuan et al. focused on the importance of a larger dataset for better generalization to unseen data. In their experience, a dataset of 1593 produced a near-perfect overall score and lower BBB−. The same experiment has been repeated on a larger dataset of 1990 compounds, for which the classifier accuracy dropped from 98.7% to 95.7%.

Shen et al. (2010) and Wang et al. have achieved high sensitivity scores of 99.6%, and 99.0%, respectively. Both studies failed to greatly improve the specificity, obtaining 85.7% and 83.3%, respectively. The results of these studies show the importance of improving the classifier training process, so that it performs just as well on unseen data. They also demonstrate that more attention should be focused on obtaining consistent predictions for both class labels.

Martins et al. (2012) presented a comparative study of SVM and random forest (RF) on a larger dataset of 1970 compounds, achieving a high specificity score of 96%. However, the high specificity score came at the expense of sensitivity accuracy, which reached only 58%. Recently, oversampling techniques been applied to improve the model training process (Wang et al., 2018; Zhu, Lin & Liu, 2017; Wang et al., 2019; Nakamura et al., 2013). Wang et al. (2018) tested multiple resampling methods to improve the prediction of the BBB−class. They presented an empirical comparative analysis of different oversampling methods for the problem of BBB permeability, including random undersampling (RUS) (Blagus & Lusa, 2013), the adaptive synthetic sampling approach(ADASYN) (He et al., 2008), weight loss function (WLF), and the synthetic minority oversampling technique (SMOTE) (Chawla et al., 2002). Their best model achieved only 83.3% when predicting compounds with low permeability. A comparison of the performance of a selection of studies from the literature is presented in Table 1.

A QSAR dataset is characterized by high dimensionality. One of the first attempts to reduce the high dimensionality of the BBB dataset was performed by Li et al. (2005b). They applied feature selection using a technique known as recursive feature elimination (RFE). They developed an SVM classification model and emphasized the positive effect of feature extraction for the model’s accuracy. One of few studies focused on generating a large set of molecular descriptors and fingerprints to improve classifier accuracy was conducted by Yuan, Zheng & Zhan (2018). They proposed an SVM model trained with 1875 (1D, 2D, and 3D) descriptors and five types of fingerprints, and obtained an overall accuracy of 93.96% and a specificity score of 91%. Their model was the first to achieve such a good score in both class labels. They reported that a subset featuring fingerprints outperformed the set containing molecular descriptors. Alsenan, Al-Turaiki & Hafez (2020) explored the effect of applying a non-linear feature extraction method to reduce the high dimensionality of a BBB dataset. A high-dimensional dataset composed of 6,394 molecular descriptors and fingerprints was generated to accomplish this task. Their study was based on a neural network technique known as an autoencoder. They compared their proposed approach to PCA and concluded that non-linear techniques outperformed linear extraction methods in overall accuracy and in separating class labels in a binary classification QSAR problem.

Various ligand-based BBB studies have contributed tremendously to the prediction of compounds with high BBB permeability. However, predicting compounds with low permeability has become a consistently challenging task for researchers. More investigation is encouraged to identifying means of improving the minority class to achieve better classification. A recent shift in QSAR studies has been directed toward developing DL methods to solve various QSAR problems (Lo et al., 2018; Thomas et al., 2020). Research in BBB permeability with DL approaches is still in its infancy, and many variations of DL models have yet to be investigated.

## Research Methodology

To solve the three issues identified in the BBB permeability problem, related to dataset, algorithm and features, we present an enhanced FFDNN model and a CNN model that address each of these shortcomings.

**Table 1:** Classification accuracy measures of the studies reported from the literature.

Model	Test set					
	Evaluation	ACC	Sens.	Spec.	AUC	MCC
SVM (Wang et al., 2018)	10-fold	91.2	92.5	89.9	90.8	–
Consensus MLP (Wang et al., 2018)	10-fold	96.6	99	83.3	91.9	–
DT (Martins et al., 2012)	5-fold	92.22	0.58	0.96	–	0.55
SVM (Li et al., 2005a; Li et al., 2005b)	5-fold	83.7	88.6	75.0	–	0.65
ANN (Guerra, Páez & Campillo, 2008)	LGO	73.0	68.0	80.0	–	0.79
DT (Castillo-Garit et al., 2017)	10-fold	87.93	86.67	89.29	–	–
SVM (Yuan, Zheng & Zhan, 2018)	70/30+ 5-fold	93.96	94.3 91.0	94.3 91.0	–	0.84

**Notes.**

ACC Overall accuracy Sens Sensitivity scores Spec Specificity scores MCC Matheow correlation coefficient AUC Area under the curves

**Figure 1:**
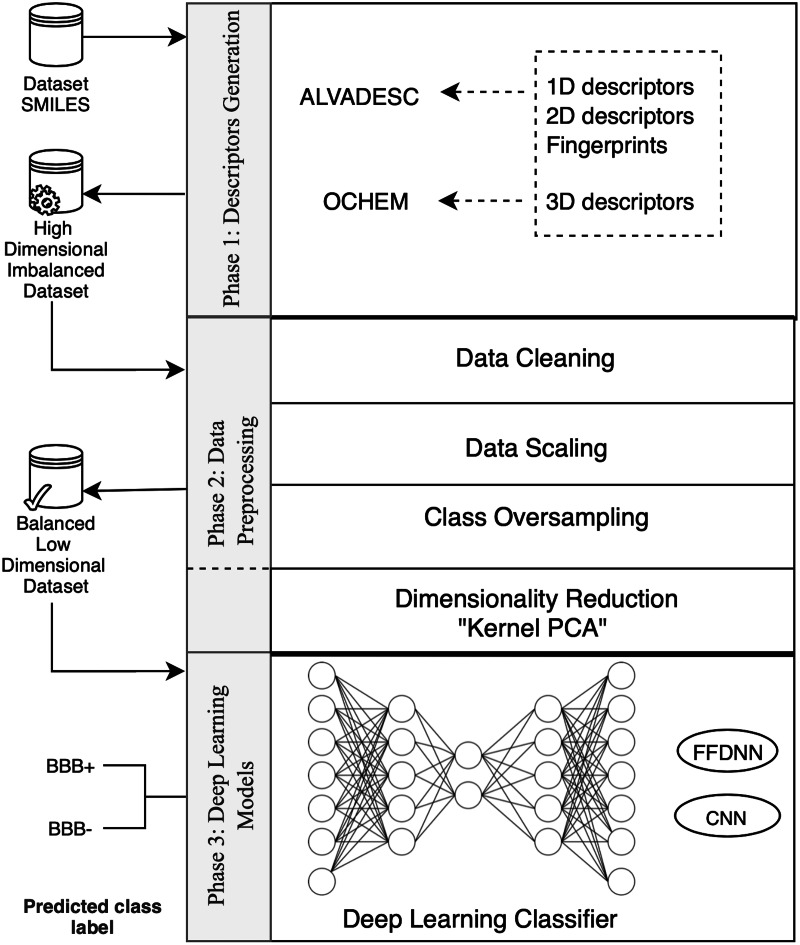
The four phases of developing the BBB permeability model.

The proposed model is composed of three main phases, illustrated in Fig. 1. In the first phase, we generate a full set of molecular descriptors and fingerprints. The output of this step is an imbalanced high-dimensional BBB dataset composed of 6,394 descriptors and 2,500 records. The second phase involves all the data preprocessing tasks required to produce a clean, balanced dataset. This includes handling empty records, dealing with outliers, data scaling, and oversampling. Additionally, this phase uses KPCA to transform the dataset from a high- to a low-dimensional Euclidean space. The third phase involves the comparative empirical development of an FFDNN model and a CNN model. The models are tested individually with the dataset produced in phase 2. Each model’s output is the predicted class labels, representing compounds classified as either BBB+ or BBB−. The proposed models are assessed based on five accuracy measures; accuracy, specificity, sensitivity, area under the curve (AUC), and Matthew Correlation Coefficient (MCC), to identify the best DL approach to predict BBB permeability.

### Data description

Developing a model based on a benchmark dataset enables the comparison of the model’s performance to other studies from the literature. Wang et al. (2018) gathered a dataset of 2,350 compounds obtained from previous line of BBB permeability studies, which focused on the compounds’ penetration into the BBB through passive diffusion. The dataset comprised 1,803 compounds representing the positive class (BBB+) and 547 compounds representing the negative class (BBB−). In QSAR modelling, compounds in the form of 1D, 2D, and 3D structures are transformed into canonical simple linear notations, such as Simplified Molecular Input Line Entry Specifications (SMILES) (Xu & Hagler, 2002). All compounds in the dataset were encoded into SMILES representations. The criteria for dividing compounds into the BBB+ class or BBB- class were taken from previous studies and the benchmark study by Wang et al. (2018). Compounds are classified as BBB+ or BBB−when Log BB value is > = −1 and when the Log BB value is < − 1, respectively.

### Data analysis

To ensure a reliable prediction of a QSAR model, applicability domain (AD) should be defined by specifying the model’s limitation. The AD accounts the prediction of external compounds as reliable if they fall within the defined domain’s scope. Hence, AD determines how well the proposed models can generalize to unseen data outside the training set (Sheridan et al., 2004). AD can be determined by many statistics-based approaches to analyze the descriptors space. We used distance-based method to ensure testing compounds are structurally similar to those in the training set. The molecular weight (MW), TPSA, and ALogP descriptors were considered for the applicability domain analysis.

**Figure 2:**
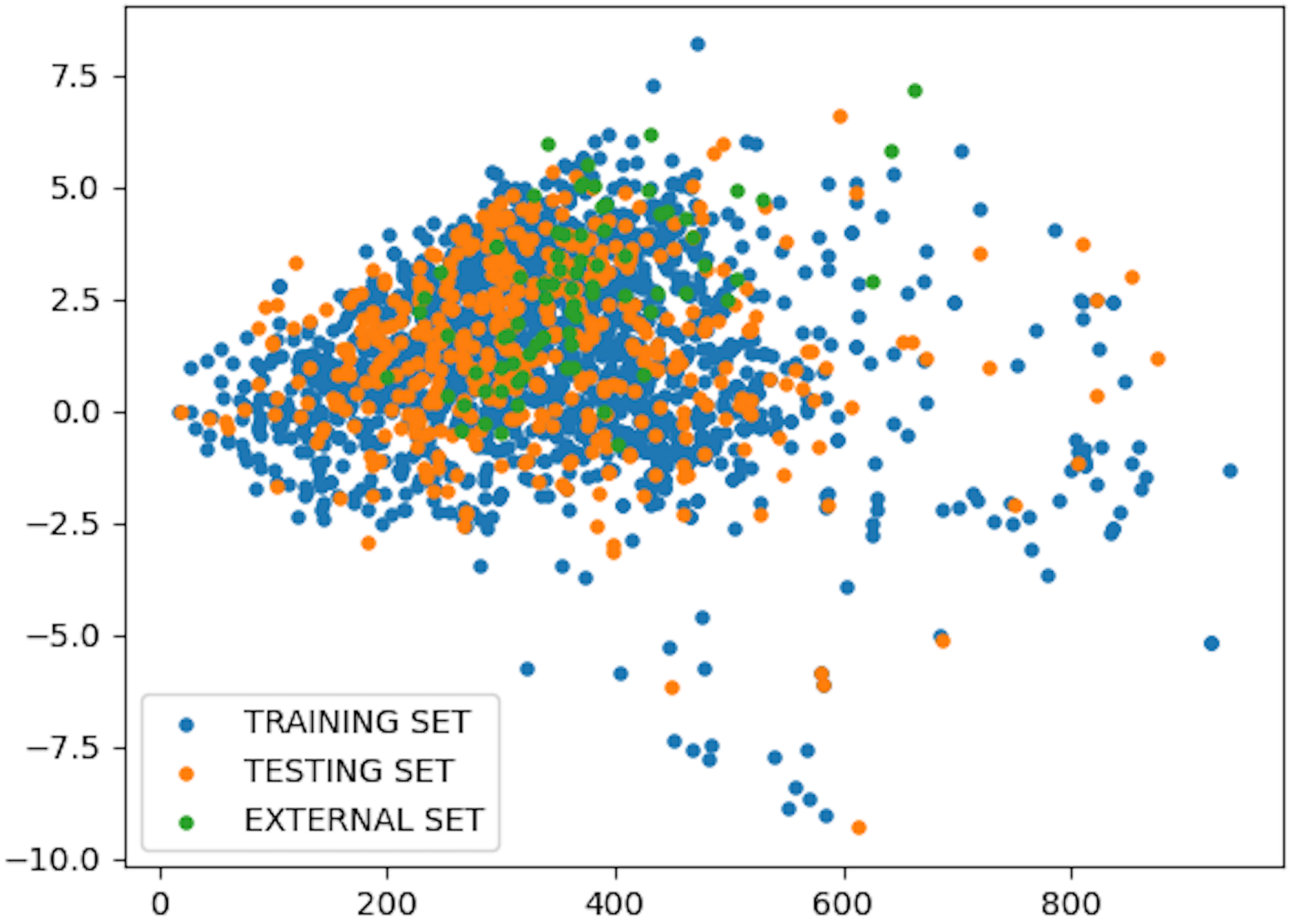
Dataset analysis.

The average Euclidean distance distance between each compound in the dataset is 745.08 for the aforementioned descriptors. The mean of MW is 338.22, ALogP is 1.412, and TPSA is 114.96. The standard deviation for MW is 135.96, for ALogP is 2.028, and for TPSA is 83.18. If the new query point average distance vary from the value defined above for these features its prediction will be unreliable. Figure 2 is a scatter plot demonstrating the chemical space distribution of the training, testing and external datsaet with respect to descriptors MW and ALogP. Despite the high range, the three datasets are positioned within the same chemical space.

### Descriptors calculation

To produce the best classifier performance with respect to molecular descriptors and fingerprints, a high-dimensional dataset was created with 6,394 descriptors and fingerprints calculated using the alvaDesc application (Alvascience Srl, 2019) and Ochem platform (Sushko et al., 2011). The molecular descriptors included 0D descriptors, such as constitutional indices, bond counts, weight, and atom counts; 2D descriptors based on molecular topology, such as topological indices and fragment counts; and 3D descriptors, such as gravitational index, charged partial surface area (CPSA), and weighted holistic invariant molecular (WHIM). For each SMILES record, 6,394 descriptors and fingerprints were calculated.

Ultimately, the generated descriptors made up a high-dimensional space, which was inputted to the KPCA technique to extract the descriptors with the most value for the classification task. To load SMILES onto the program, the CSV file was converted to SMIformat. For the purposes of our study, all descriptor types were selected and 3,874 1D and 2D descriptors were calculated. The Ochem platform used the Chemistry Development Kit (CDK) (Steinbeck et al., 2003), and the partial charge and atom coordinates of 3D descriptors were obtained with a BALLOONoptimizer. Of the 2,350 records, 2,342 were successfully calculated for the 1D, 2D, and 3D descriptors. AlvaDesc was used to calculate two types of fingerprints: Hashed and MACCS 166. MACCS are fixed-size 2D fingerprints consisting of 166 bits, and they represent the most important features of a molecule. The appearance or absence of each of the 166 bit represents specific, well-defined features. Hashed fingerprints are Boolean vectors that describe the molecule structure in every possible pattern (also called exhaustive patterns). Hashed fingerprints are processed with a hashing function to reduce them to a fixed, specific size.

Because hashed fingerprints describe every possible pattern of a molecule structure, they have the advantage of being able to provide a larger range of descriptions of the molecule’s structure. There are two main types of hashed fingerprints: extended connectivity fingerprints (ECFP) and path fingerprints (Danishuddin & Khan, 2016). In a Python environment, the dataset was saved and read as comma-separated values, known as a CSV file. Panda library reads CSV files as dataframes, which can easily manipulate and process data by selecting columns, replacing values, retrieving rows, etc. Once 1D, 2D, and 3D descriptors were calculated, all columns in the dataframe were concatenated using Panda library so that each SMILES record in the dataset contained 1D, 2D, and 3D descriptors and fingerprints. The final number of attributes (descriptors) in the dataset was 6,394. *The supporting file* provides a summary of the total number of descriptors, their dimensions, and the tool used.

### Data preprocessing

Once all descriptors and fingerprints were calculated for each SMILES record in the dataset, preprocessing was performed on the dataset. Proper preprocessing results in clean, correct, and complete data while improper preprocessing may lead to poor results and affect the accuracy and efficiency of the algorithm (Li et al., 2005a).

#### Data cleaning

During the descriptors calculation process, 2,342 of 2,350 compounds were successfully calculated. Eight compound descriptors were not obtained by Ochem because their atom coordinates and atomic partial charges were not calculated. Those records had null values for all descriptors. Using Panda library in Python, these SMILES records were located and dropped. In addition, some SMILES records had some successfully calculated descriptors but some missing values. There are multiple ways to handle missing values, such as replacing them by zero, replacing them by a mean value, or imputation. We computed the mean value of each column and replaced all missing values by the calculated mean value of their respective columns.

#### Scaling (data transformation)

After data cleaning was performed, the dataset was ready for normalizing as it had a varying range of values. MinMax scaler was needed to normalize the descriptors’ range (Juszczak, Tax & Duin, 2002). The values of 1D, 2D, and 3D descriptors were transformed using the MinMax scaler to a range between 0 and 1. To apply the scaler, we first fit the MinMax scaler on the training data and transformed the external validation dataset using the trained scaler.

#### Handling class imbalance

The dataset obtained from Wang et al. (2018) was imbalanced in terms of class distribution. As shown in the literature, previous BBB studies have struggled to obtain high classification scores with respect to the negative class because class imbalance was not properly addressed. Based on Wang et al. and a comparative analysis of several oversampling techniques reported in the literature, we adopted an oversampling technique proposed by Chawla et al. (2002) known as the synthetic minority oversampling technique (SMOTE), which delivers consistent, adequate classification accuracy. Several studies have proven the positive effect of oversampling techniques on the classifiers ability to learn, including Wang et al. (2018), Chawla et al. (2002), López, Fernández & Herrera (2014), and Nguyen, Cooper & Kamei (2011). Synthesizing new instances by proximity through oversampling does not necessarily mean that the new instances are real, but rather allows the classifier to generalize.

SMOTE focuses on improving the minority class by means of oversampling or on improving the majority class by undersampling, depending on the dataset (Chawla et al., 2002). It uses a *k*-nearest neighbors approach, which increases the number of data points (instances) in the minority class by creating new ones. The original data points remain unchanged, but they are used as input to synthesize (produce) new ones along with their respective features. The features’ values are estimated by generating new samples with features from data points in the target class (in our case, BBB−) and their neighbors.

#### Dimensionality reduction

Once the data was cleaned, scaled, and integrated into one file, it was ready for dimensionality reduction. This step is critical to developing a classification model. High-dimensional data causes noise and redundancy and increases computational complexity (Danishuddin & Khan, 2016). It was apparent from the literature that feature selection methods were widely used with QSAR modeling because they are effective at removing redundant features prior to applying feature extraction techniques (Khalid, Khalil & Nasreen, 2014; Meng et al., 2018). The most-used feature extraction technique was *linear principal component analysis* (PCA) (Goodarzi, Heyden & Funar-Timofei, 2013). However, multiple studies argued that non-linear extraction methods are superior to selection methods in extracting complex structures and hidden patterns (Pirhadi, Shiri & Ghasemi, 2015). KPCA is a variation of linear PCA capable of learning nonlinear representations of data.

Suppose we have D dimensional vectors that we want to project to a dimension space M. Given a dataset *x*_*i*_, where *i* = 1, 2, 3, ..., *N*, and *x*_*i*_ is the D dimensional vector, the data points *x*_*i*_ can be transformed to a nonlinear representation denoted as Φ(*x*).

There are multiple parameters for the PCA kernel, linear, sigmoid, cosine, polynomial, and radial basis functions (Mushtaq et al., 2019). The choice of kernel function for feature extraction purposes is based on the data itself. Experimenting with multiple kernels can provide insight on which kernel to use (Ustun, 2009). Linear kernel was not tested due to its slow processing; it might be more suitable for linear data (Ezukwoke, 2019).

The number of components returned by KPCA can be specified, but our model allowed KPCA to return the best set of descriptors with non-zero values in the reduced dimension space. This was been accomplished by not passing any value to KPCA for the parameter on the number of components “n_components”, which means that all non-zero values were returned. This allowed our model to retain all valuable information. After passing the complete high-dimensional dataset to KPCA, 3,603 descriptors were returned.

Based on the performance of kernels, we used polynomial kernel for the FFDNN model and Cosine kernel for the CNN model. For fast computation, the number of jobs running in parallel was set to 10 jobs. The number of descriptors was transformed from 6,394 to 3,603 descriptors. The processed, balanced, cleaned and lower dimensional data was then used as an input to the deep learning models. In addition, both models used the same set of descriptors generated from KPCA.

#### Performance measures

Five accuracy measures are generally used across BBB permeability studies and QSAR research: accuracy, specificity, sensitivity, area under the curve (AUC), and the Matthews correlation coefficient (MCC). Accuracy indicates the overall performance of the model, but it is not regarded as a good indicator of the model’s performance due to the class imbalance in BBB permeability datasets. Specificity is the percentage of compounds correctly classified as BBB−by the model, and sensitivity is the percentage of compounds correctly classified as BBB+ by the model.

(1) \begin{eqnarray*}Accuracy= \frac{TP+TN}{TP+TN+FP+FN} \end{eqnarray*}

(2) \begin{eqnarray*}Specificity= \frac{TN}{TN+FP} \end{eqnarray*}

(3) \begin{eqnarray*}Sensitivity= \frac{TP}{TP+FN} \end{eqnarray*}

(4) \begin{eqnarray*}MCC= \frac{(TP\times TN)-(FP\times FN)}{\sqrt{(FP+TN)(FP+TP)(FN+TN)(FN+TP)}} \end{eqnarray*}

Another means of comparing the performance of the proposed classifiers is using AUC to assess how well the classifier separates classes by calculating the area under the ROC curve. MCC is a measure commonly used in QSAR, especially in imbalanced binary classification. Both measures are used to measures the proposed models’ performance (Fawcett, 2006).

To validate the model and measure its predictive ability, we followed the best practices for QSAR predictive modeling (Tropsha, 2010) by validating the model with independent external validation. In addition, the model was validated with *K*-fold cross-validation. As for *K*-fold cross-validation, the *K* value was 10, and the original dataset was portioned into 10 subsets (folds). Of the 10 subsets, one was retained for testing while the remaining subsets were used for the training task. This process was repeated until each subset was used for testing once. An advantage of this technique is that it works on small datasets and can reveal the occurrence of overfitting. According to Arlot & Celisse (2010), the estimate bias with 10-fold cross-validation is minimal in classification tasks. To minimize bias, the session was cleared after each fold to avoid overfitting caused by retaining neurons weights. This was accomplished by invoking a *clear*_*session()* command after each fold. Once all the training and testing with K-fold validation was finished, we tested the model with an external dataset obtained from Drugbank (http://www.drugbank.ca/) (Drugbank, 2005) comprising 86 CNS+. We excluded 8 (BBB−) instances from the original dataset to be tested as part of the external dataset.

## Deep Learning Classification Models

This research aims to develop a classification model to predict BBB permeability. We investigated and conducted a comparative experiment with two deep learning models to tackle the problem of BBB permeability. Previous BBB models were developed with shallow ANN or FFDNN models. Collectively, they either achieved low accuracy in predicting compounds’ permeability (Guerra, Páez & Campillo, 2008; Li et al., 2005b) or achieved a higher accuracy score for one measure than the other (Wang et al., 2018; Martins et al., 2012). We first presented an enhanced FFDNN to overcome the difficutly of predicting compounds with low permeability, classified as BBB−. Then, we explored a CNN model to investigate its capabilities in handling the complexity of a BBB dataset. The enhanced FFDNN and CNN models were developed using a Python PyCharm environment. The models were developed with an imported dataset retrieved by SMOTE, consisting of 3,606 compounds. The number of epochs was set to 100, and the batch size was set to 200. A fixed training/testing validation, a 10-fold cross-validation, and an external validation were performed to test the models’ performance. The sample information for the experiments is presented in Table 2.

**Table 2:** Details of the training, testing, and external datasets used in this study.

Sample size		**Training set**	Testing set	External set	Descriptors	Descriptors
Before SMOTE	After SMOTE				before KPCA	after KPCA
1803 BBB+	1803 BBB+	1,874	468	86	6,394	3,603
547 BBB−	1803 BBB−					

### Enhanced FFDNN model

Building a DL model can be challenging. The FFDNN model was enhanced by three main contributions: applying a resampling method to improve the class distribution of the imbalanced dataset, transforming the dataset with a KPCA technique to improve the predictive power of the model, and experimentally optimizing the FFDNN model by tuning the hyper-parameters.

To optimize and tune a network, multiple hyper-parameter choices ought to be explored. Deep learning hyper-parameters is tuning the network parameters, such as learning rate, weights, and number of neurons and layers, to reach the optimal learning potential (Snoek et al., 2015). We experimentally considered multiple activation functions, optimizers, and validation techniques. The final list of hyper-parameters to optimize the FFDNN model is summarized in the *Supporting Information*.

For the enhanced FFDNN model, we present a five-layer DL network. These layers include an input layer, three hidden layers, and an output layer. In addition, two batch normalization layers are used. Batch normalization is a technique that separates neural networks’ layers to help stabilize the layers’ distribution and adjust the activation functions. This is accomplished by taking the output of each layer and normalizing it before it is inputted to the next layer and by calculating the mean and the variance of the input (Santurkar et al., 2018).

**Figure 3:**
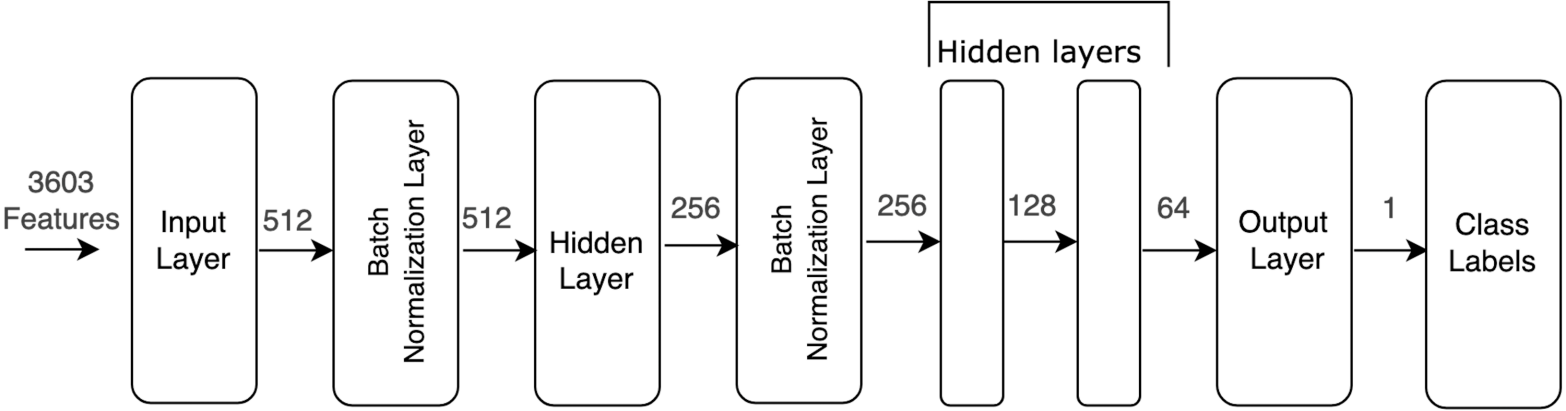
Block diagram of FFDNN model.

To choose the activation functions, we experimented with a combination of rectified linear units (ReLU) and Tanh. By applying ReLU in the first input layer and Tanh on the remaining layers, we achieved better model learning and less computational cost. Three optimizers were tested for the model: adaptive moment estimation (Adam), stochastic gradient descent (SGD), and rectified adaptive Moment estimation (RAadm). Because of its adaptive rate, Adam showed the best performance when fitting the model. The proposed model contained three hidden layers (also called dense layers). The number of neurons in each layer is Dense(512), Dense(256), Dense(128), Dense(64), Dense(32), and Dense(1). Batch normalization was applied after two layers, as shown in Fig. 3. Because BBB classification is a binary problem, a sigmoid function was applied to the last layer to predict the class label, assigned as 1 or 0 for BBB+ or BBB−, respectively.

### Convolutional Neural Network (CNN) model

**Figure 4:**
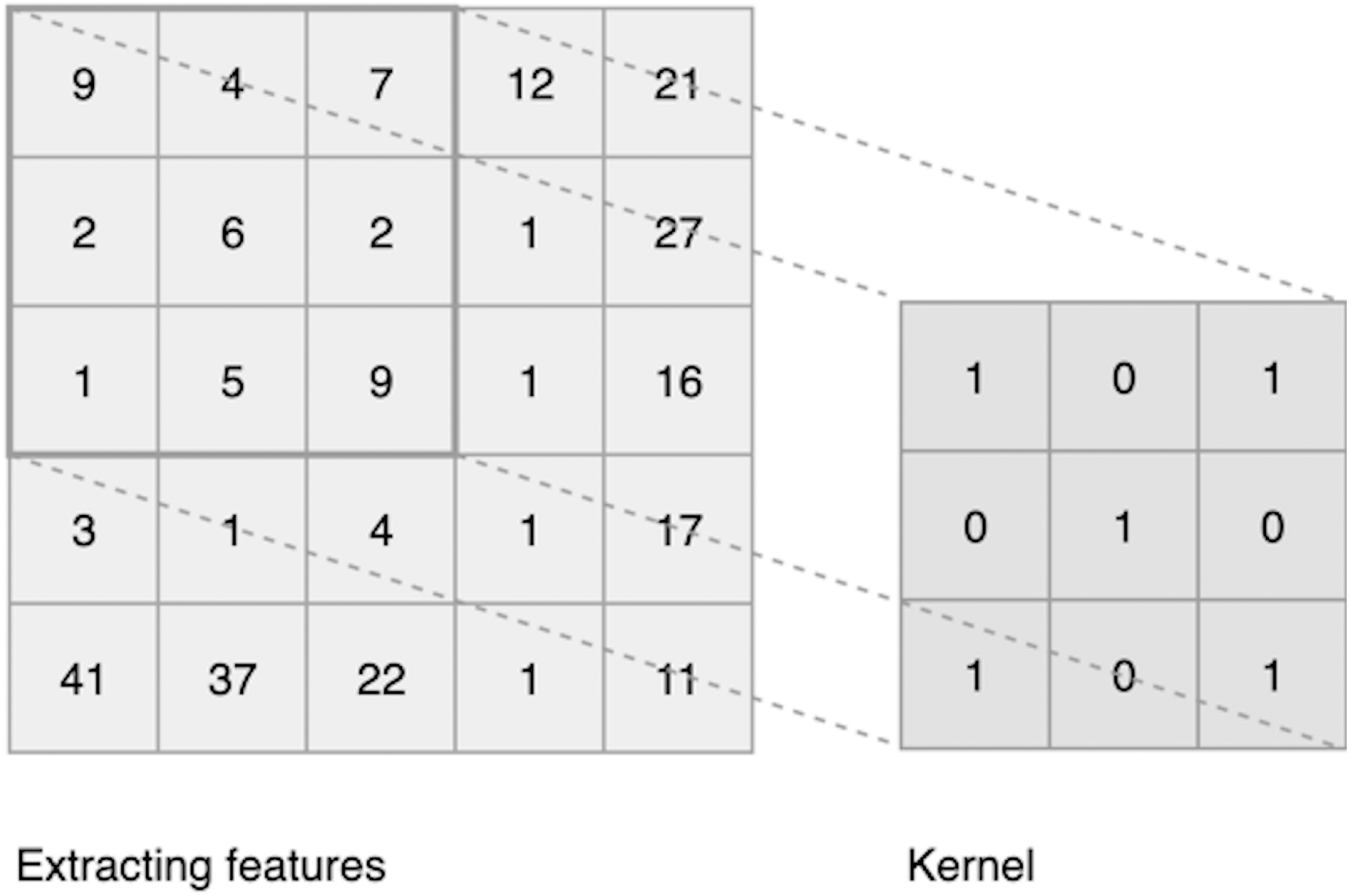
Convolutional layer.

Convolutional Neural Networks (CNN) share similar characteristics as feed-forward neural networks in the aspects of being constructed from neurons, weights, and biases. They are also similar in the process of calculating weights and activation functions. CNN is a special type of feed forward neural networks that are known for their state of the art results in visual representations and images processing (Schmidhuber, 2015). CNN differs from a typical DNN in the structure of the hidden layers. CNN architecture is typically composed of three layers; convolutional layers, pooling layers and fully connected layers. These layers are formed in 3 dimensions: height, width, and depth. The convolution is a mathematical algorithm that takes an input (such as an image) and extract features using the kernel to produce an output. CNN works in “windows” known as “kernels”. Kernels use shared parameters or filters in a form of a 3 by 3 matrix. It works by sliding these parameters over an input to calculate features. The extracted features are known as a “feature map” or “convolved feature”, as shown in Fig. 4.

The pooling layers reduce the dimensionality of a feature map by picking up the features with highest value, or the most important features. The pooling layer has multiple advantages. First, it makes any input, whether visual or textual representation, smaller. Second, it reduces the number of features (dimensionality). The convolution and pooling layers are the fundamental base of CNNs. The convolution and pooling processes are repeated because the input to a second convolution layer is the output of the first pooling layer. Finally, the output is fed to the fully connected layer, which is a normal multilayer perceptron (FFDNN) with an output layer that uses softmax (Chen et al., 2018).

**Figure 5:**
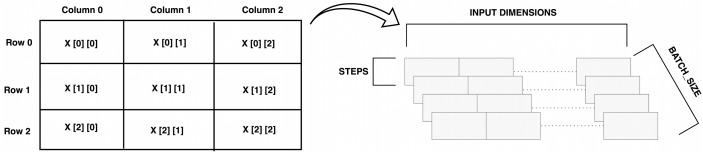
Transforming network shape from 2D to 3D.

Two common types of convolutions are used with CNN models, Conv2D function which is employed mostly with images, and Conv1D which is used with sequences and textual problems. Because the problem in hand is textual Conv1D is used. To input the dataset to the CNN model several steps were taken. The current dataset is in a 2D shape that is processed with two parameters (*batch*_*size*, *input*_*dim*) where the *batch*_*size* represents the number of samples in one iteration and the *input*_*dim* refers to the number of descriptors (features) in the dataset. The CNN layers implemented in this model is from tensorflow (keras) library that requires a 3D tensor data in the form: (*batch*_*size*, *steps*, *input*_*dim*), where the steps are needed to add another dimension to represent the depth of the array. The process of converting the data from 2D to 3D shape is illustrated in Fig. 5 where the matrix on the left denote the original dataset. The array element X[0][0] represents the first instance encoded in (SMILES) representation and the first descriptor, X[0][1] represents the array position of the same instance with the second descriptor in the array and so on. When the array is inputted to the CNN a “steps” parameter was added to represent the third dimension of the CNN architecture

**Figure 6:**
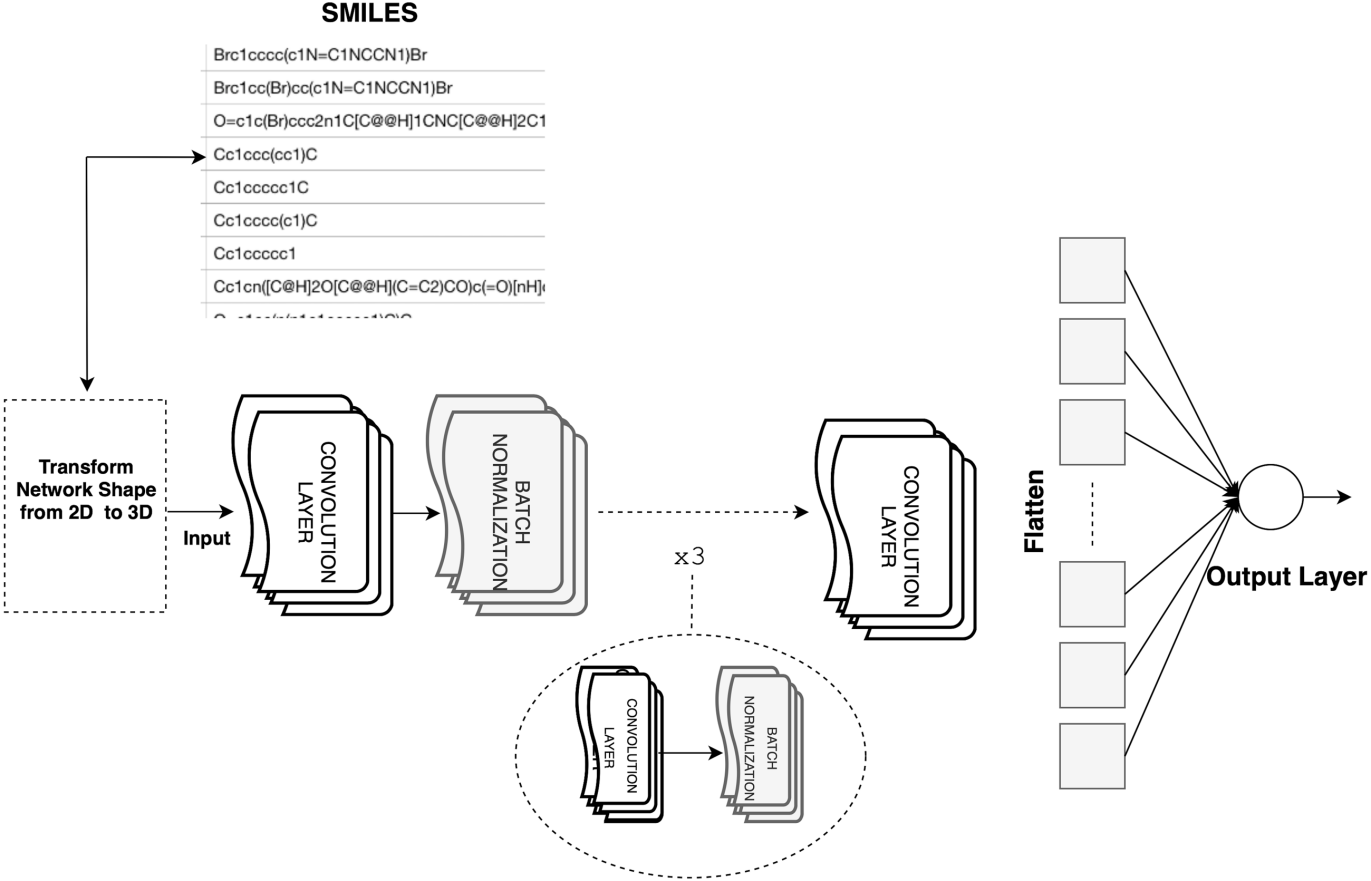
CNN architecture.

The dataset comprises 2,350 chemical compounds encoded into SMILES representation along with their descriptors and fingerprints. To construct the CNN model, our dataset had to be modified to a 3D shape. Each convolution layer uses filters to calculate feature maps. Filters are the parameters in which the CNN learns. For the CNN architecture, we used four convolution layers, four batch normalization layers, one flattened layer, and one dense layer, as shown in Fig. 6. We set the activation function to ReLU and the optimizer to Adam. The number of neurons for each hidden unit was set to (1024, 512, 512, 256, 1). The model was fitted for training and then for generating the class prediction.

## Results

Measuring how well a model performs with respect to real data is an essential task when building a QSAR classification model. In this section, we present the performance analysis of the proposed models. First, we present the experimental results of the proposed DL models. We report the results of the enhanced FFDNN model and the CNN model. Model progression in response to different hyperparameters’ tuning is presented in the Supporting Information. To further investigate the ability of the FFDNN model and CNN model in identifying the class labels, they are compared with some of the most widely used ML models, namely SVM, random forest, and XGboost. In addition, the data transformation after employing KPCA and SMOTE is demonstrated.

### Deep learning models results

We experimented the aforementioned activation functions, optimizers, and other hyper-parameters. The *Supporting File* list the results of different hyper-parameters on the network performance. Initially, both ReLU and Leaky ReLU failed to identify the negative class. In the first run, ReLU suffered from the dying ReLU problem and failed to classify any compound with low penetration (BBB−). The second experiment was conducted with a combination of *“Tanh and ReLU”*, which resulted in a enhanced performance of the FFDNN compared with just ReLU on the FFDNN. Batch normalization and the MinMax scaler contributed to normalizing the layers and regularizing the range of descriptors’ values. As for the Adam optimizer, the adaptive learning rate of weights showed an apparent improvement, as the overall accuracy increased from 75.95% to 91.06% with SGD. However, the specificity scores still showed varying results between training and testing, as shown in the first row of Table 3, prior to employing SMOTE. The training set in the best model reached a specificity of 99.33% compared to 83.35% during testing, which indicates overfitting issues.

**Table 3:** SMOTE effect on the enhanced FFDNN model.

Resampling (SMOTE)	Training set			Test set					
	ACC	Sens.	Spec.	ACC	Sens.	Spec.	AUC	MCC	CI(95%)
No SMOTE	99.78	99.93	99.33	91.06	93.04	83.35	92.00	73.68	.057–.125
SMOTE *K* = 9	99.86	99.88	99.77	96.17	93.72	98.61	98.61	92.46	.033–.081
SMOTE *K* = 12	99.89	99.79	100	96.20	93.51	98.89	98.73	92.54	.032–.082
SMOTE *K* = 12 + KPCA	100	96.78	98.11	97.11	97.35	98.42	99.50	95.55	.020–.072

**Figure 7:**
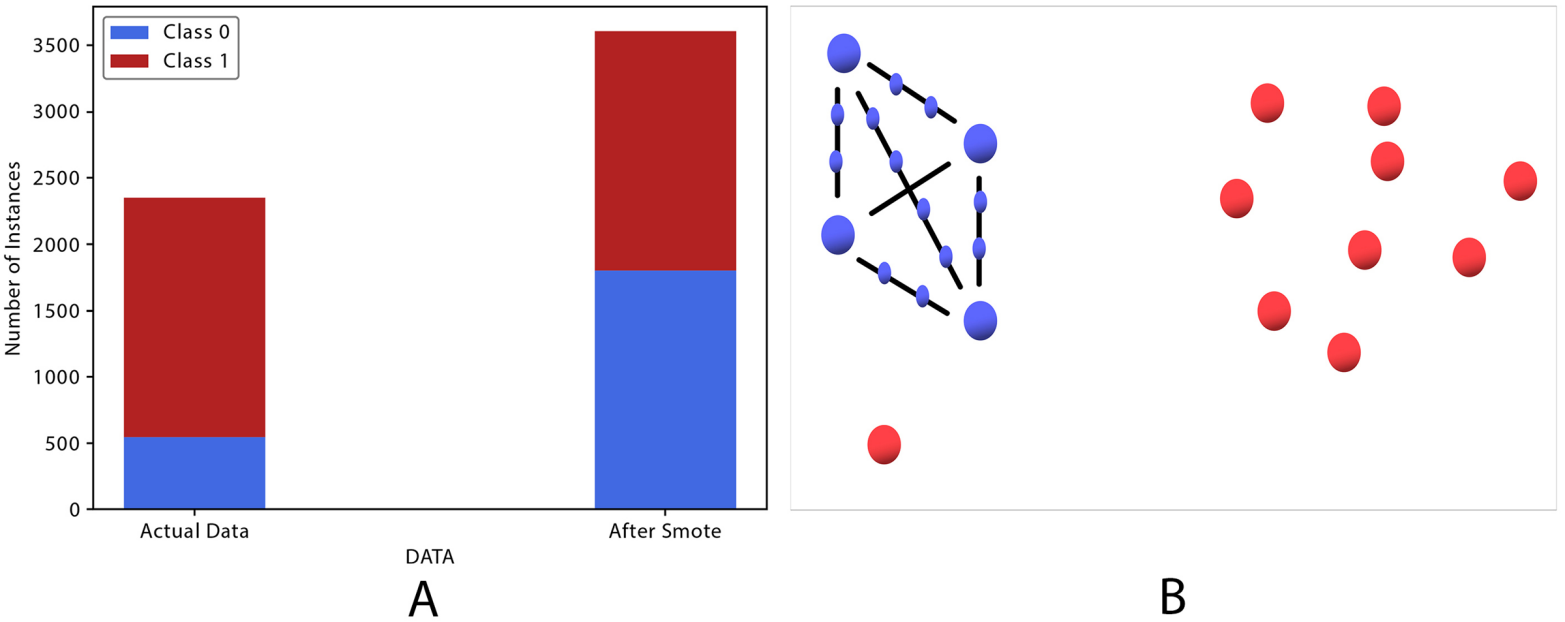
SMOTE oversampling technique. (A) Class labels transformation. (B) Synthesizing new instance.

To address the model’s low predictive ability in the negative class (BBB−), a SMOTE resampling technique was used (Nakamura et al., 2013). SMOTE demonstrated remarkable enhancement of the negative class; the specificity results improved from 83.35 to 98.61, as shown in Table 3. SMOTE transformed the dataset from 2,350 compounds, 1,803 and 547 in the positive and negative classes, respectively, to 1,803 compounds in each class. In our proposed models, SMOTE created new points in such a way that all synthesized points are on the line between two original minority points as illustrated in Fig. 7B. This figure represents the majority class with red big points and the minority class with big blue points. Because we have many red data points and only four blue points, SMOTE was used to create new small blue points located between the original instances. This SMOTE resampling technique transformed the data set from 1,803 and 547 compounds in the positive and negative classes, respectively, to 1,803 compounds in each class, as shown in Fig. 7A.

**Figure 8:**
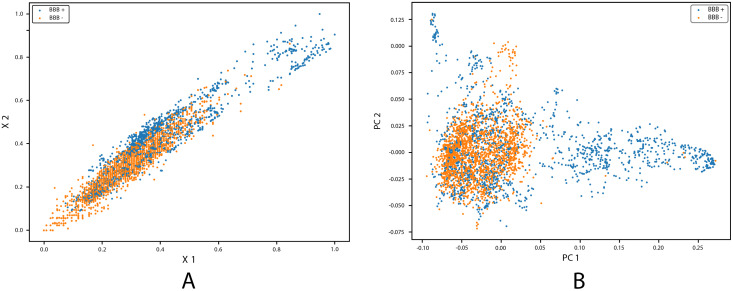
Dataset transformation with Kernel PCA. (A) Original dataset. (B) After kernel PCA.

To further understand how the distribution of the dataset was transformed after employing KPCA, we used visual encoding to visualize the data distribution. We designed a scatter plot of the BBB dataset before and after using KPCA. As shown in Figs. 8A and 8B, the data points in the original dataset are positively correlated with respect to two features. This is an undesirable quality that indicates redundancy in the feature space and does not necessarily improve the model’s learning. Figure 8B shows the transformation of the data-points with respect to PC1 and PC2 after the application of KPCA. KPCA was able to separate and scatter compounds with respect to PC1 and PC2, achieving low positive correlation. The classes were clearly separable even before being inputted into the classification model. KPCA transformed the high-dimensional datset to a low dimensional space and improved the classifier’s ability to separate classes. The enhanced FFDNN with SMOTE and KPCA achieved an overall accuracy of 97.11%, a specificity score of 98.42%, and a sensitivity score of 97.35% on the testing set.

The CNN model was inputted a balanced dataset delivered by the SMOTE oversampling technique. The high-dimensional dataset was reduced with KPCA. The models fitted during training delivered a near perfect score, which shows they learned well during the training process. The CNN model achieved an overall score of 97.76% with 10-fold validation. In predicting compounds that penetrate the BBB, the best CNN model achieved a sensitivity score of 94.50% and a specificity score of 98.31%. The obtained area under the curve of the CNN model was 99.71%. As seen in Table 4, the classification accuracy with an external dataset was 0.97 overall with the CNN model and 0.965 with FFDNN.

**Table 4:** Performance evaluation of the proposed DL models in comparison with ML methods and benchmark.

Model	Training set			Test set					
	ACC	Sens.	Spec.	ACC	Sens.	Spec.	AUC	MCC	ACC-Ext
FFDNN	100	96.78	98.11	97.11	97.35	98.42	97.7	95.55	96.5
CNN	100	98.76	99.87	97.76	94.50	98.31	99.71	92.85	97.0
XGBoost	98.67	96.23	92.34	94.32	92.34	95.66	93.22	83.44	92.00
SVM	99.32	98.30	95.62	95.94	95.30	96.62	93.3	93.92	93.90
RF	99.47	90.21	97.15	93.61	90.21	97.15	93.68	87.46	92.04

**Notes.**

ACC Overall accuracy Sens Sensitivity scores Spec Specificity scores MCC Matheow correlation coefficient AUC Area under the curves ACC-Ext Overall accuracy on external dataset

Table 4 summarizes the performance of the CNN and FFDNN models using 10-fold cross-validation and an independent external validation. For the dimensionality reduction task, different kernels were tested. Cosine and polynomial kernel extracted the best features for the CNN and the FFDNN models, respectively. The CNN model achieved an overall accuracy of 97.76%, a sensitivity score of 94.5% and a specificity score of 98.31%.The FFDNN model achieved an overall accuracy of 97.11%, sensitivity value of 97.35%, and specificity value of 98.42%. Following Tropsha’s (2010) best practices for validating a QSAR predictive model, an independent external dataset was acquired from Drugbank (http://www.drugbank.ca/) (Drugbank, 2005) with 86 BBB+ instances. The overall accuracy achieved on the external dataset was 97.0% for the CNN model and 96.5% for the FFDNN model.

### Comparative results: DL vs. ML models

To validate the performance of the proposed deep learning approach in comparison with traditional machine learning models, we run few experiments with three widely used machine learning models. We run the experiments under the same configuration and environment to ensure a fare comparison. The same descriptors set extracted by KPCA is used for the deep learning and machine learning models. We also compare our results with the benchmark study by Wang et al. (2018).

Three additional ML models were developed: XGboost, SVM, and RF. For the SVM model, the main goal of the kernel was to separate classes in the higher dimension. A non-linear sigmoid kernel was employed. SVM achieved an overall accuracy of 95.94, a sensitivity of 95.30%, and a specificity of 96.62%. XGboost achieved an overall accuracy of 94.32, a sensitivity of 92.34%, and a specificity of 95.66%. RF generates an ensemble of decision trees, which is usually trained with a “bagging method”. RF employs randomness when searching for features among a random subset of features, which ensures diversity in choosing features. The number of estimators was set to 10, which indicates the number of trees in the forest. The maximum feature parameter was set to “auto”. RF scored 93.61% in overall accuracy, 90.21% in sensitivity, and 97.15%in specificity.

XGBoost is an ensemble learning algorithm that stands for extreme gradient boosting. Ensemble learning has the advantage of using the prediction capabilities of multiple learning models. XGBoost uses a gradient descent algorithm to minimize loss when adding new models (Wang, Deng & Wang, 2020). Parameters such as the tree booster and number of features were set to default. The number of threads was set to the maximum available. XGboost achieved an overall accuracy of 94.32%, a sensitivity of 92.34%, and a specificity of 95.66%.

**Figure 9:**
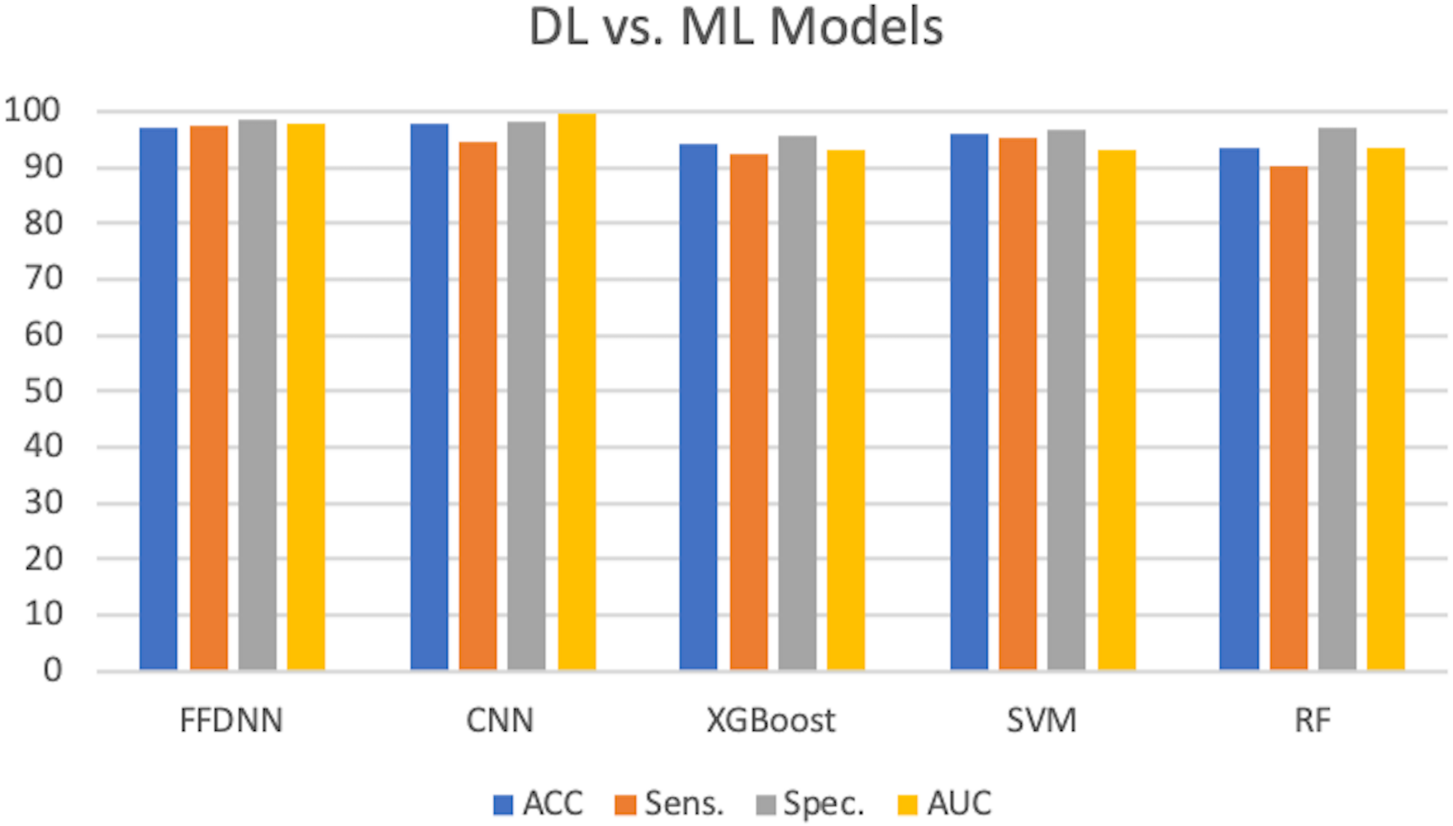
DL vs. ML models.

**Figure 10:**
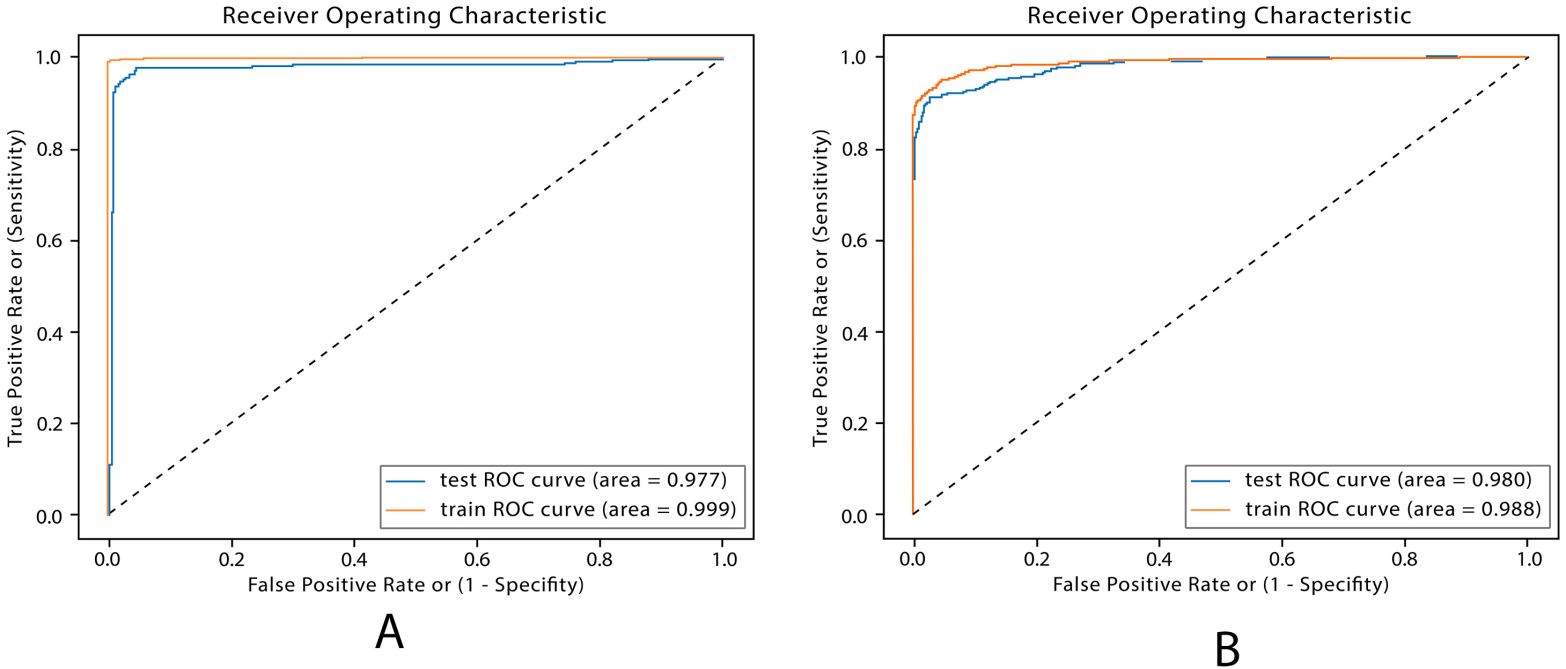
ROC plots for DL models. (A) ROC Enhanced FFDNN. (B) ROC CNN.

Figure 9 illustrated the comparison between the deep learning approach with the traditional machine learning models. However, the FFDNN and CNN models outperformed the three studied models in all accuracy measures.

AUC is used in validating the performance of binary classification models. AUC shows the classifiers ability to separate compounds classified as BBB+ or BBB−. The closest to the top left corner near 1, the more the model is capable of separating the two class labels. The proposed DL models achieved an AUC score of 98.6% for the FFDNN model and 98.9% for the CNN model. The ROC plots of the DL models are shown in Fig. 10, with the FFDNN ROC demonstrating the best performance. The ROC graphs are plotted near the top left corner, which indicates a better balance between sensitivity and specificity.

**Figure 11:**
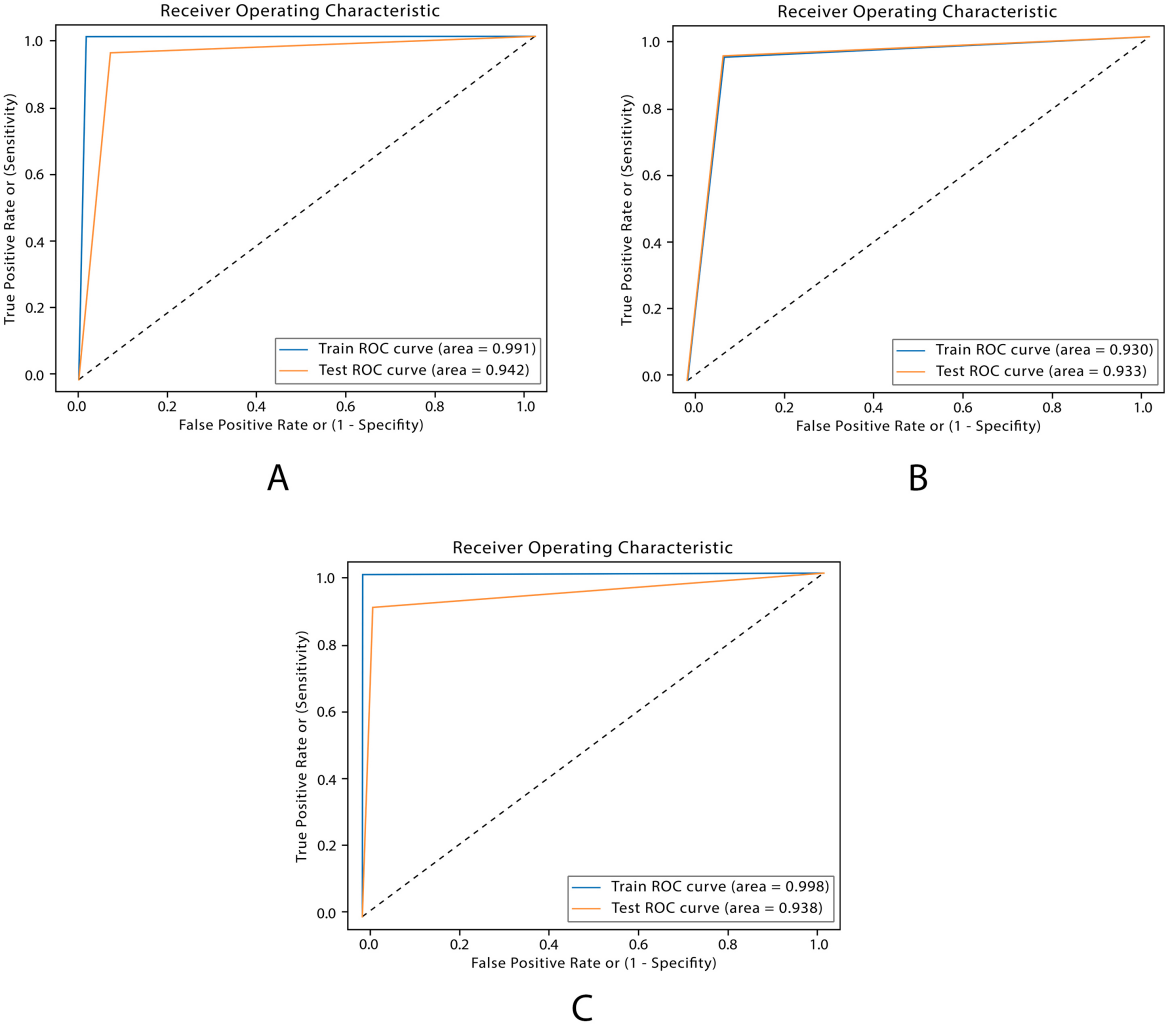
ROC plots for ML models. (A) ROC XGboost. (B) ROC SVM. C) ROC RF.

The AUC scores of SVM, XGBoost and RF models are 93.3%, 94.2% and 93.8%, respectively as shown in Fig. 11. SVM demonstrated similar AUC score as RF and XGBoost although it scored a higher overall accuracy. This is an indication of a high rate of false positive.

## Discussion

In this section, we discuss the main finding of the proposed enhanced FFDNN model and the CNN model. delivered adequate results. Many steps were taken to prepare the data for the experiments including employing SMOTE and KPCA. Based on the results obtained by our FFDNN and CNN models, several valuable observations have been made. Employing KPCA significantly affected the classifiers performance positively as many redundant descriptors were removed and only valuable ones were retained. Compared with other feature extraction and feature selection methods from the literature, KPCA delivered adequate results.

The SMOTE resampling technique has been tested in previous BBB permeability studies using the same benchmark dataset. However, the benchmark study didn’t reach a specificity score higher than 89.9% (Wang et al., 2018). As for the proposed DL models, SMOTE was able to transform the imbalanced BBB dataset by oversampling the minority class. Although the duplicated records from the minority class did not provide new information, they preserved the model’s ability to predict both class labels. SMOTE achieved a specificity value of 98.42% and 98.31% for the FFDNN and CNN models, respectively. This confirms that the right hyper-parameters for the classification model have great influence on its performance. SMOTE and KPCA played an essential role in predicting the negative class (i.e., compounds with low permeability) without compromising the accuracy scores when predicting the positive class. The proposed models therefore achieved high accuracy across all measures, which indicates a promising lead in BBB classification research.

By a large margin, the FFDNN and CNN models outperformed those used in all previous BBB permeability studies in the literature in terms of specificity score. The proposed DL models reached a higher overall accuracy than the benchmark dataset by Wang et al. (2018). However, evaluating the model’s performance with unseen data provides some insight into its ability to generalize to new data and highlights any signs of overfitting. The proposed CNN model achieved a higher overall accuracy score of 97.00% on the external dataset, compared to that by Wang et al. (2018). Both DL models obtained higher accuracy, specificity, and sensitivity scores compared with other ML algorithms in the literature, such as decision trees and SVM models. Of the three ML models, SVM achieved the most consistent scores and the highest accuracy, sensitivity, and AUC. When comparing the CNN and FFDNN models, the CNN achieved the highest overall accuracy but the FFDNN achieved the best balance of sensitivity and specificity.

One of the main issues this study aimed to investigate is how to enhance the prediction of compounds with low permeability. The proposed enhanced FFDNN and CNN models reached specificity scores of 98.42% and 98.31%, respectively. The gained improvement in specificity scores achieves one of the study’s main goals which is to minimize high false positive rates. False positives occur when the model achieves high BBB+ predictions accuracy only without regard to the falsely classified positives. The proposed DL models were consistent in predicting BBB+ and BBB−classes compared to SVM, RF, and XGboost models. The obtained area under the curve of the CNN model indicates that the model was successful in separating the two class labels based on the learned features. The CNN model was able to outperform the best consensus model by Wang et al. (2018), which was computed by adding extra compounds from DrugBank, without the addition of extra compounds from DrugBank.

## Conclusion

BBB permeability is a complex problem that requires a deep understanding of various dataset characteristics. The construction of the proposed DL models focused on addressing three main issues: class imbalance, high dimensionality, and high false positive rates resulting from high sensitivity and low specificity scores. This research shed light on the importance of resolving issues present in a BBB dataset before applying the classification task, with a specific focus on exploring a DL approach to solve the BBB permeability problem.

This study contributes to the body of knowledge by addressing a significant gap in many BBB permeability models, which is the ability to predict compounds with low permeability. This reoccurring issue is overlooked, resulting in high false positive rates. The proposed DL models delivered consistently high scores in all measures and surpassed state-of-the-art models, such as SVM, RF, and XGboost, in terms of specificity scores and overall accuracy.

We encourage future studies to investigate other non-linear dimensionality reduction techniques able to fully exploit the different characteristics of QSAR descriptors and fingerprints. Future studies may also focus on projecting back feature extraction techniques to identify which descriptors were highly influential to the prediction task.

##  Supplemental Information

10.7717/peerj-cs.515/supp-1 Supplemental Information 1 Supporting information for the deep learning approach

## Additional Information and Declarations

### Data Availability

The following information was supplied regarding data availability: Data and code are available at GitHub: https://github.com/S-A-A-BBB/BBB-Prediction.git.

## References

[ref-1] Alsenan S, Al-Turaiki I, Hafez A (2020). Autoencoder-based dimensionality reduction for QSAR modeling.

[ref-2] Alvascience Srl (2019). alvaDesc. https://www.alvascience.com.

[ref-3] Arlot S, Celisse A (2010). A survey of cross-validation procedures for model selection. Statistics Surveys.

[ref-4] Bagchi S, Chhibber T, Lahooti B, Verma A, Borse V, Jayant RD (2019). In-vitro blood-brain barrier models for drug screening and permeation studies: an overview. Drug Design, Development and Therapy.

[ref-5] Blagus R, Lusa L (2013). Improved shrunken centroid classifiers for high-dimensional class-imbalanced data. BMC Bioinformatics.

[ref-6] Bradbury MW (1993). The blood-brain barrier. Experimental Physiology: Translation and Integration.

[ref-7] Brito-Sánchez Y, Marrero-Ponce Y, Barigye SJ, Yaber-Goenaga I, Morellérez C, Le-Thi-Thu H, Cherkasov A (2015). Towards better BBB passage prediction using an extensive and curated data set. Molecular Informatics.

[ref-8] Carpenter TS, Kirshner DA, Lau EY, Wong SE, Nilmeier JP, Lightstone FC (2014). A method to predict blood-brain barrier permeability of drug-like compounds using molecular dynamics simulations. Biophysical Journal.

[ref-9] Castillo-Garit JA, Casanola-Martin GM, Le-Thi-Thu H, Pham-The H, Barigye SJ (2017). A simple method to predict blood-brain barrier permeability of drug- like compounds using classification trees. Medicinal Chemistry.

[ref-10] Chawla NV, Bowyer KW, Hall LO, Kegelmeyer WP (2002). SMOTE: synthetic minority over-sampling technique. International Journal of Artificial Intelligence Research.

[ref-11] Chen H, Engkvist O, Wang Y, Olivecrona M, Blaschke T (2018). The rise of deep learning in drug discovery. Drug Discovery Today.

[ref-12] Chevalier R (2018). Penetration assessment of dietary supplements and drugs through the blood-brain barrier for potential treatment of parkinson’s disease.

[ref-13] Danishuddin, Khan AU (2016). Descriptors and their selection methods in QSAR analysis: paradigm for drug design. Drug Discovery Today.

[ref-14] Desai BS, Monahan AJ, Carvey PM, Hendey B (2007). Blood–brain barrier pathology in Alzheimer’s and Parkinson’s disease: implications for drug therapy. Cell Transplantation.

[ref-15] Dorronsoro I, Chana A, Abasolo MI, Castro A, Gil C, Stud M, Martinez A (2004). CODES/neural network model: a useful tool for in silico prediction of oral absorption and blood-brain barrier permeability of structurally diverse drugs. J. QSAR COMB. SCI..

[ref-16] Drugbank (2005). Open Data Drug and Drug Target database, moxifloxacin. DB00218. https://go.drugbank.com/drugs.

[ref-17] Ezukwoke K (2019). Kernel methods for principal component analysis (PCA) A comparative study of classical and kernel PCA. A preprint.

[ref-18] Fawcett T (2006). An introduction to ROC analysis. Pattern Recognition Letters.

[ref-19] Fukushima K, Wake N (1991). Handwritten alphanumeric character recognition by the neocognitron. IEEE Transactions on Neural Networks and Learning Systems.

[ref-20] Gao Z, Chen Y, Cai X, Xu R (2017). Predict drug permeability to blood-brain-barrier from clinical phenotypes: drug side effects and drug indications. Bioinformatics.

[ref-21] Garg P, Verma J (2006). In silico prediction of blood brain barrier permeability: an artificial neural network model. Journal of Chemical Information and Modeling.

[ref-22] Ghaddar B, Naoum-Sawaya J (2018). High dimensional data classification and feature selection using support vector machines. European Journal of Operational Research.

[ref-23] Goodarzi M, Heyden YV, Funar-Timofei S (2013). Towards better understanding of feature-selection or reduction techniques for Quantitative StructureActivity Relationship models. Trends in Analytical Chemistry.

[ref-24] Guerra A, Páez JA, Campillo NE (2008). Artificial neural networks in admet modeling: prediction of bloodbrain barrier permeation. QSAR & Combinatorial Science.

[ref-25] He H, Bai Y, Garcia EA, Li S (2008). ADASYN: Adaptive synthetic sampling approach for imbalanced learning.

[ref-26] Juszczak P, Tax D, Duin RP (2002). Feature scaling in support vector data description. http://rduin.nl/papers/asci_02_occ.pdf.

[ref-27] Khalid S, Khalil T, Nasreen S (2014).

[ref-28] Kortagere S, Chekmarev D, Welsh WJ, Ekins S (2008). New predictive models for bloodbrain barrier permeability of drug-like molecules. Pharmaceutical Research.

[ref-29] Li D, Deogun J, Spaulding W, Shuart B (2005a). Dealing with missing data: algorithms based on fuzzy set and rough set theories. Transactions on rough sets IV.

[ref-30] Li H, Yap CW, Ung CY, Xue Y, Cao ZW, Chen YZ (2005b). Effect of selection of molecular descriptors on the prediction of bloodbrain barrier penetrating and nonpenetrating agents by statistical learning methods. Journal of Chemical Information and Modeling.

[ref-31] Lo Y-C, Rensi SE, Torng W, Altman RB (2018). Machine learning in chemoinformatics and drug discovery. Drug Discovery Today.

[ref-32] López V, Fernández A, Herrera F (2014). On the importance of the validation technique for classification with imbalanced datasets: addressing covariate shift when data is skewed. Journal of Information Science.

[ref-33] Ma J, Sheridan RP, Liaw A, Dahl GE, Svetnik V (2015). Deep Neural Nets as a Method for Quantitative StructureActivity Relationships. Journal of Chemical Information and Modeling.

[ref-34] Martins IF, Teixeira AL, Pinheiro L, Falcao AO (2012). A bayesian approach to in silico blood-brain barrier penetration modeling. Journal of Chemical Information and Modeling.

[ref-35] Meng Q, Catchpoole D, Skillicorn D, Kennedy PJ (2018). Relational autoencoder for feature extraction.

[ref-36] Miao R, Xia L-Y, Chen H-H, Huang H-H, Liang Y (2019). Improved classification of blood-brain-barrier drugs using deep learning. Scientific Reports.

[ref-37] Mushtaq Z, Yaqub A, Hassan A, Su SF (2019). Performance analysis of supervised classifiers using PCA based techniques on breast cancer.

[ref-38] Nakamura M, Kajiwara Y, Otsuka A, Kimura H (2013). Lvq-smote–learning vector quantization based synthetic minority over–sampling technique for biomedical data. BioData Mining.

[ref-39] Nguyen HM, Cooper EW, Kamei K (2011). Borderline over-sampling for imbalanced data classification. International Journal of Knowledge Engineering and Soft Data Paradigms.

[ref-40] Pardridge WM (1999). Blood-brain barrier biology and methodology. Journal of Neurovirology.

[ref-41] Pirhadi S, Shiri F, Ghasemi JB (2015). Multivariate statistical analysis methods in QSAR. RSC Advances.

[ref-42] Santurkar S, Tsipras D, Ilyas A, Madry A (2018). How does batch normalization help optimization?. 32nd Conference on Neural Information Processing Systems (NIPS 2018).

[ref-43] Schmidhuber J (2015). Deep learning in neural networks: an overview. Neural Networks.

[ref-44] Seddon AM, Casey D, Law RV, Gee A, Templer RH, Ces O (2009). Drug interactions with lipid membranes. Chemical Society Reviews.

[ref-45] Shen J, Cheng F, Xu Y, Li W, Tang Y (2010). Estimation of ADME properties with substructure pattern recognition. Journal of Chemical Information and Modeling.

[ref-46] Sheridan RP, Feuston BP, Maiorov VN, Kearsley SK (2004). Similarity to molecules in the training set is a good discriminator for prediction accuracy in QSAR. Journal of Chemical Information and Computer Sciences.

[ref-47] Snoek J, Rippel O, Swersky K, Kiros R, Satish N, Sundaram N, Patwary M, Prabhat M, Adams R (2015). Scalable bayesian optimization using deep neural networks.

[ref-48] Steinbeck C, Han Y, Kuhn S, Horlacher O, Luttmann E, Willighagen E (2003). The Chemistry Development Kit (CDK): an open-source Java library for chemo-and bioinformatics. Journal of Chemical Information and Computer Sciences.

[ref-49] Suenderhauf C, Hammann F, Huwyler J (2012). Computational prediction of blood-brain barrier permeability using decision tree induction. Molecules.

[ref-50] Sushko I, Novotarskyi S, Körner R, Pandey AK, Rupp M, Teetz W, Brandmaier S, Abdelaziz A, Prokopenko VV, Tanchuk VY (2011). Online chemical modeling environment (OCHEM): web platform for data storage, model development and publishing of chemical information. Journal of Computer-Aided Molecular Design.

[ref-51] Thomas JJ, Tran THN, Lechuga GP, Belaton B (2020). Convolutional graph neural networks: A review and applications of graph autoencoder in chemoinformatics. Deep learning techniques and optimization strategies in big data analytics.

[ref-52] Tropsha A (2010). Best practices for QSAR model development, validation, and exploitation. Molecular Informatics.

[ref-53] Ustun B (2009). Support vector machines: facilitating the interpretation and application. https://repository.ubn.ru.nl/handle/2066/75822.

[ref-54] Wang C, Deng C, Wang S (2020). Imbalance-XGBoost: leveraging weighted and focal losses for binary label-imbalanced classification with XGBoost. Pattern Recognition Letters.

[ref-55] Wang X, Yu B, Ma A, Chen C, Liu B, Ma Q (2019). Protein–protein interaction sites prediction by ensemble random forests with synthetic minority oversampling technique. Bioinformatics.

[ref-56] Wang Z, Yang H, Wu Z, Wang T, Li W, Tang Y, Liu G (2018). In silico prediction of blood-brain barrier permeability of compounds by machine learning and resampling methods. ChemMedChem.

[ref-57] Wermuth CG, Villoutreix B, Grisoni S, Olivier A, Rocher J-P, Wermuth CG, Aldous D, Raboisson P, Rognan D (2015). Chapter 4—strategies in the search for new lead compounds or original working hypotheses.

[ref-58] Xu J, Hagler A (2002). Chemoinformatics and drug discovery. Molecules.

[ref-59] Yuan Y, Zheng F, Zhan C.-G (2018). Improved prediction of blood-brain barrier permeability through machine learning with combined use of molecular property-based descriptors and fingerprints. The AAPS Journal.

[ref-60] Zhang L, Zhu H, Oprea TI, Golbraikh A, Tropsha A (2008). QSAR modeling of the blood-brain barrier permeability for diverse organic compounds. Pharmaceutical Research.

[ref-61] Zhu T, Lin Y, Liu Y (2017). Synthetic minority oversampling technique for multiclass imbalance problems. Pattern Recognition.

