# Supplementary material for: A deep learning approach to predict blood-brain barrier permeability
Source: PeerJ Comput Sci. 2021 Jun 10;7:e515. doi: 10.7717/peerj-cs.515 (PMC8205267; doi:10.7717/peerj-cs.515)
Supplement: Supplemental Information 1 [file peerj-cs-07-515-s001.pdf]

1 DEEP LEARNING OVERVIEW SUPPORTING INFORMATION

1.1 Activation Functions

1. **Sigmoid:**

$$F(X) = \frac{1}{1+e^{\left(-\sum_j w_j x_j - b\right)}} \tag{1}$$

Simplified to:

$$f(X) = \frac{1}{1+e^{-x}} \tag{2}$$

The shape of sigmoid function is shown in 1(b).

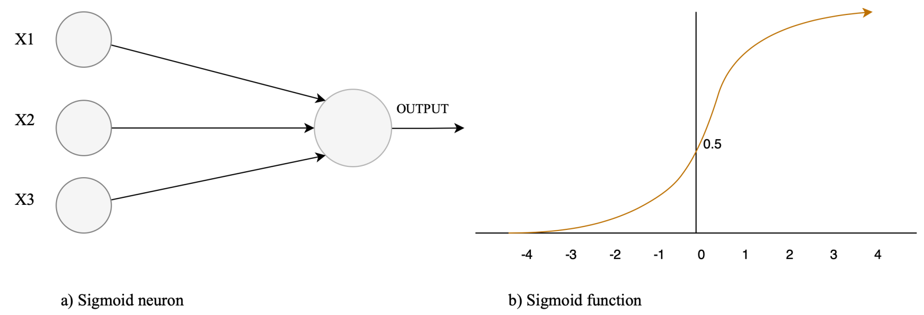

Figure S1: Sigmoid function

2. **ReLU and Leaky ReLU:**

$$ReLU(x) = \frac{0 if x < 0}{x if x > 0} \tag{3}$$

This equation can be written as:

$$f(x) = max(0, x) \tag{4}$$

Leaky ReLU is defined as:

$$f(x) = max(0.01, x) \tag{5}$$

3. **Tanh: hyperbolic tangent:**

$$f(x) = tanh(x) = \frac{2}{1+e^{-2x}} - 1 \tag{6}$$

1.2 MinMax scaler equation

MinMax scaler can be calculated as shown in the following equation:

$$X_{norm} = \frac{X - X_{min}}{X_{max} - X_{min}} \tag{7}$$

Table S1: Final number of attributes (descriptors

Descriptor type	Number of descriptors	Tool	Final Count
1D and 2D	3874	Alvadesc (noa)	
3D	306	Ochem (3)	
MACCS	166	Alvadesc (noa)	6394 descriptors
Hashed (ECFP)	1024	Alvadesc (noa)	
Hashed (Path)	1024	Alvadesc (noa)	

1.3 Outliers

Eight compounds descriptors failed to be calculated. Those records had null values for all descriptors. The remaining eight compounds are outliers from other descriptors pairs in the dataset as illustrated in Figure 3 and 4.

Using Panda library in Python, these SMILES records were located and dropped. The list of the dropped SMILES is show below:

- `Cc1cc(nnc1NCCN1CCOCC1)=C1C=CC(=O)C=C1`
- `O=C1C=CC=C\C1=c1\nnco1`
- `CCCC1(C)COB(OC1)C1=CC=C(C)C=C1`
- `[Kr]`
- `[Ne]`
- `[Ar]`
- `[Xe]`
- `[Rn]`

1.4 Descriptors calculation

The final number of attributes (descriptors) in our dataset is 6394 as shown in Table S1.

1.5 Validation measures

Four main accuracy measures are used across BBB permeability studies and QSAR research in general, namely: Accuracy, Specificity, Sensitivity and Mathew Correlation Coefficient (MCC).

$$Accuracy = \frac{TP+TN}{TP+TN+FP+FN} \tag{8}$$

$$Specificity = \frac{TN}{TN+FP} \tag{9}$$

$$Sensitivity = \frac{TP}{TP+FN} \tag{10}$$

$$MCC = \frac{(TP \times TN)\text{-}(FP \times FN)}{\sqrt{(FP+TN)(FP+TP)(FN+TN)(FN+TP)}} \tag{11}$$

Where TP is true positive of the number of compounds correctly classified as "positive" or BBB+; TN is true negative of compounds correctly classified as BBB- by the classifier; FP is false positive which indicate number of compounds mistakenly classified as BBB+; and FN is false negative which is number of compounds that penetrate BBB but mistakenly classified as BBB-.

1.5.1 Results of Baseline FFDNN Model

Prior to scaling the network or applying a resampling technique, we experimentally tested all the combinations of hyper-parameter tuning. The initial results of the different activation functions on the FFDNN model is presented in Table S3. The final hyperparameters set is shown in Table S3.

Table S4 demonstrates the effect of the right regularizing and tuning of the model in the overall performance.

2 DATASET

A downloadable link to the dataset can be obtained from (Git): https://github.com/S-A-A-BBB/BBB-Prediction.git.

REFERENCES

[noa] Alvascience Srl, alvaDesc (software for molecular descriptors calculation, visited: 2019-10-16).

[Git] Github, septermber 2020 https://github.com/s-a-a-bbb/bbb-prediction.git.

[3] Sushko, I., Novotarskyi, S., Körner, R., Pandey, A. K., Rupp, M., Teetz, W., Brandmaier, S., Abdelaziz, A., Prokopenko, V. V., Tanchuk, V. Y., et al. (2011). Online chemical modeling environment (ochem): web platform for data storage, model development and publishing of chemical information. J. Comput. Aided Mol., 25(6):533–554.

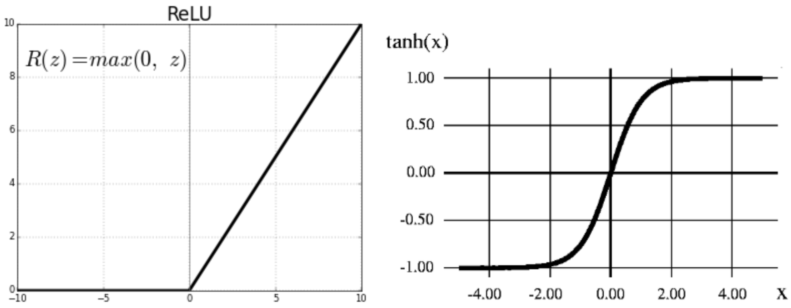

(a) ReLU **(b)** Tanh

Figure S2: ReLU and Tanh

Table S2: Model hyper-parameter

**hyper-parameter**	**Value**
Number of hidden layers	3
Number of hidden layers nodes	256,128,64
Activation function	Input layer: ReLU<br>Tanh
Batch size	200
Number of epochs	100
Optimizer	Adam
Regularization	2 layers of Batch Normalization
Scaler	MinMax scaler
Learning rate	0.01
Validation	10 fold cross validation
Loss	Binary crossentropy
Resampling technique	SMOTE
Feature extraction	Kernel PCA

Table S3: FFDNN with with different Activation Functions
(Act. Function= Activation Function, Sens= Sensitivity scores, Spec= Specificity scores, ACC= Overall accuracy, MCC= Matheow correlation coefficient, AUC= Area under the curves).

Activation	Training set			Test set				
Function	Acc	Sens.	Spec.	Acc	Sens.	Spec.	ROC	MCC
RelU	76.96	100.0	0.0	75.74	100.0	0.0	50.0	0
Tanh	83.45	95.39	45.19	86.17	95.94	50.0	81.50	54.47
LeakyRelu	76.59	100.0	0.0	77.23	100.0	0.0	50.0	0.0
Tanh+ ReLU	82.18	92.96	47.29	80.63	91.28	42.71	76.20	38.20

Table S4: FFDNN with different Optimizers

Model	Training set			Test set				
(optimizer)	ACC	Sens.	Spec.	ACC	Sens.	Spec.	ROC	MCC
Tanh + Adam	99.78	99.79	99.77	91.21	94.69	80.35	94.46	75.75
Tanh + SGD	80.79	81.70	77.69	77.65	77.936	76.85	86.41	49.8
ReLU+ Tanh + Adam	99.78	99.93	99.33	91.06	93.04	83.35	92.00	73.68
ReLU+ Tanh + SGD	82.92	82.51	84.30	75.95	77.71	70.85	81.92	44.47

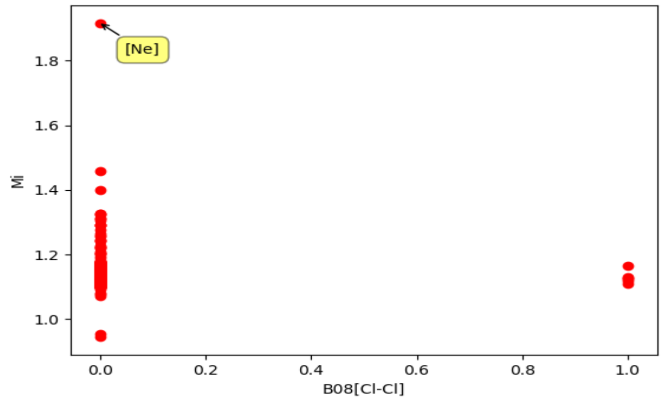

Figure S3: Outier 1

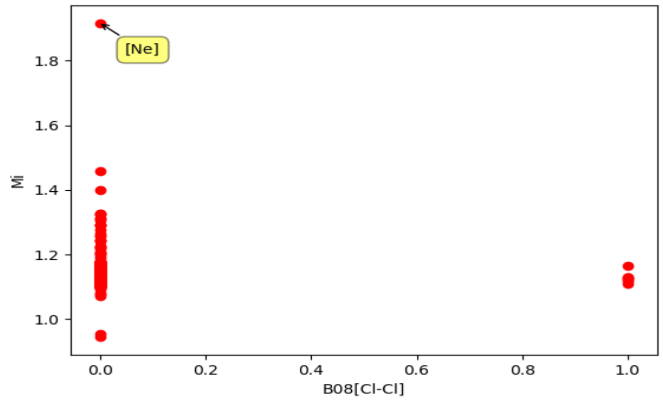

Figure S4: Outier 2

Table S5: FFDNN with SMOTE

Act. Func.	SMOTE Num of K	Training set ACC	Sens.	Spec.	Test set ACC	Sens.	Spec.	ROC	MCC
Tanh	9	99.86	99.82	99.97	95.86	93.26	98.42	98.65	91.85
	12	99.86	99.78	99.94	96.25	93.84	98.66	98.53	92.62
ReLU+ Tanh	9	99.86	99.88	99.77	96.17	93.72	98.61	98.61	92.46
	12	99.89	99.79	100	96.20	93.51	98.89	98.73	92.54

Table S6: Performance comparison of K-fold validation vs. fixed split

(ACC= Overall accuracy, Sens= Sensitivity scores, Spec= Specificity scores, MCC= Matheow correlation coefficient, AUC= Area under the curves, ACC-Ext= Overall accuracy on external dataset, Valid= Validation method).

Model	Training set Valid.	ACC	Sens.	Spec.	Test set ACC	Sens.	Spec.	AUC	MCC	CI(95%)	ACC-Ext
FFDNN	10-fold	100	96.78	98.11	97.11	97.35	98.42	97.7	95.55	.020 - .072	0.965
FFDNN	80/20	100	95.76	97.77	96.95	94.76	98.94	98.6	93.95	0.21 - 0.074	0.965
CNN	10-fold	100	98.76	99.87	97.76	94.50	98.31	98.00	92.85	.043 - .097	0.97
CNN	80-20	100	94.72	98.65	96.78	96.14	97.39	98.9	93.57	0.39 - 0.92	0.97
